# Comparing the simultaneous determination of cis- and trans-palmitoleic acid in fish oil using HPLC and GC

**DOI:** 10.1186/s12944-019-1033-4

**Published:** 2019-04-04

**Authors:** Wen-wen Huang, Bi-hong Hong, Ji-peng Sun, Ran Tan, Kai-kai Bai, Ting Yang, Hao Wu, Rui-zao Yi

**Affiliations:** 1 College of Pharmacy, Nanjing University of Chinese Medicines, Nanjing, 210023 People’s Republic of China; 2 Third Institute of Oceanography, Ministry of Natural Resources, Xiamen, 361005 People’s Republic of China; 3 Department of Hematology, Fujian Medical University Union Hospital, Fujian Provincial Key Laboratory on Hematology, Fujian Institute of Hematology, Fuzhou, 350001 People’s Republic of China

**Keywords:** Cis-palmitoleic acid, Trans-palmitoleic acid, HPLC, GC, Fish oil

## Abstract

**Background:**

Cis- and trans-palmitoleic acids (Cis-POA and trans-POA) are isomers of palmitoleic acid, a monounsaturated fatty acid which affects glucose and lipid metabolism, and reduces insulin resistance. Trans-POA is used as a biomarker for indicating the risk of type II diabetes and coronary heart disease, but no methods of analysis or distinguishing between cis-POA and trans-POA have yet been reported.

**Method:**

An accurate and precise HPLC method was developed to determine cis- and trans-POA simultaneously, and compared with results from a GC method. Cis- and trans-POA were analyzed by HPLC on a reverse-phase BDS-C18 column, equilibrated and eluted with acetonitrile (A) and water (B). In the established and validated GC method used for comparison, potassium hydroxide ester exchange was chosen to derivatize the cis- and trans-POA, before being determined.

**Results:**

The calibration curves for cis- and trans-POA were linear over the range 0.05 to 500 μg/mL. The HPLC method exhibited good sensitivity, precision and accuracy. The limits of detection (LOD) for cis- and trans-POA were 0.2 and 0.05 μg/mL, respectively. The method successfully determined cis- and trans-POA in fish oil. For the GC method, the contents of cis-POA quantified were similar to those from the HPLC method, but the contents of trans-POA revealed significant variation between the two methods.

**Conclusions:**

After a comprehensive consideration of the characteristics of the saponification and methyl esterification methods which have been tested and verified, the HPLC method was found to be suitable for determining cis- and trans-POA contents in fish oil. It was also suggested that in natural fish oil, cis-POA may be in the glyceride state, and trans-POA almost completely in the free acid form. In comparison with the GC method, the HPLC method provided a simpler process and faster analyses for identifying and determining cis- and trans-POA. The study has also provided technical support for studying the pharmacological differences and relationship between structure and activity of cis- and trans-POA. This could help physicians to analyze patients’ samples more quickly in 10 min and therefore provide a more rapid diagnosis of problems relating to the risk of type II diabetes and coronary heart disease.

## Highlights

- A rapid HPLC method was developed to identify and determine cis- and trans-POA simultaneously in 10 min
- The results for cis-POA quantified in fish oil were similar from both the HPLC and GC methods
- The contents of trans-POA revealed significant variation between the HPLC and GC methods
- Cis-POA may be in the glyceride state, with trans-POA almost completely in the free acid form in natural fish oil

## Background

**Fig. 1:**
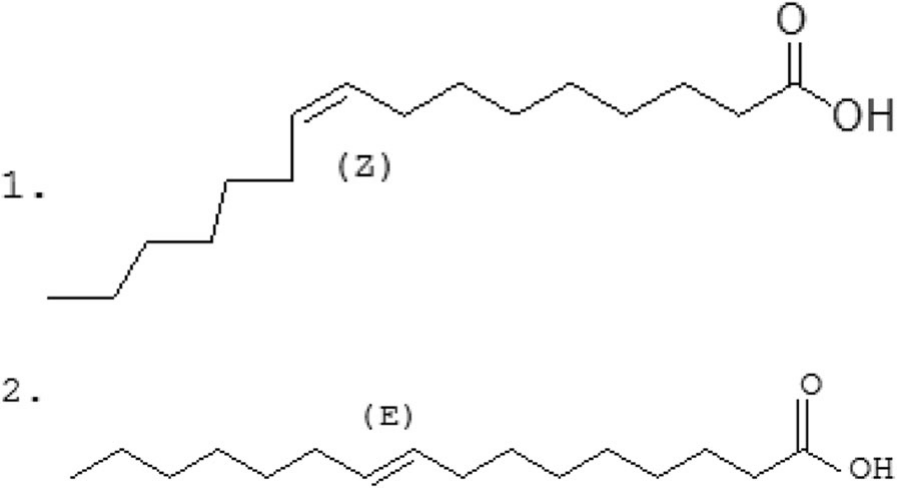
Structures of 1 - cis-palmitoleic acid and 2 - trans-palmitoleic acid

Palmitoleic acid (C16:1, n-7, POA) is a natural omega-7 monounsaturated fatty acid, which is abundant in plant and fish oils [1]. Its two isomers, cis-POA and trans-POA have different spacial structures (Fig. 1). Cis-POA is common in POA from natural sources, and has been demonstrated to favorably influence glucose and lipid metabolism through various mechanisms [2, 3]. Cis-POA affects the key enzymes during blood glucose metabolism, regulates insulin secretion in humans, and reduces insulin resistance [4, 5]. Orally-administered cis-POA has been reported to induce satiety, enhance the release of satiety hormones, and decrease the food intake in mice [6]. In mice, cis-POA also reduced body weight gain, ameliorated the development of hyperglycemia and hypertriglyceridemia, and improved insulin sensitivity [7]. However, trans-fatty acids are the subject of ongoing discussion regarding their positive and negative associations with metabolic and cardiovascular risk factors [8]. A higher proportion of trans-POA in plasma phospholipids has been shown to improve insulin sensitivity and decrease the onset of type II diabetes disease [9]. Trans-POA, which also regulates glycolipid metabolism, has been used as a biomarker for indicating the risk of type II diabetes and coronary heart disease, with a correlation between trans-POA and low-density lipoprotein being reported [10–12]. Generally, cis- and trans-POA both have therapeutic effects in chronic diseases such as diabetes, metabolic syndrome and inflammation with different mechanisms of activity and both are rich in fish oil. However, how the mechanisms of the physiological activity of cis- and trans-POA differ is not clear.

Fatty acids are usually analyzed using GC or GC-MS. The content of cis-POA was determined as 47.8% of the total fatty acids of the fruits of sea buckthorn (*Hippophae rhamnoides* L.) [13], and 6.4% of the total fatty acids of *Bupleurum Chinense DC* from China determined by GC-MS and GC-FID [14]. Usually, fatty acids, such as DHA and EPA from fish oil or other sources have been analyzed by GC or GC-MS [15–17]. Cis-palmitoleic acids can be analyzed simultaneously by common methods such as GC and GC-MS, but no method has been reported for identifying and determining cis- and trans-POA using HPLC or HPLC-MS simultaneously. Thus, developing a method for the quantitative analyses of cis- and trans-POA simultaneously is important for future pharmacological studies and applications.

Overall, this study aims to report the development, validation, and application of a rapid and sensitive method for the detection and quantitation of cis- and trans-POA in fish oil using HPLC and GC methods. Comparing these methods will reveal which method will be more suitable for determining cis- and trans-POA simultaneously in fish oil. The method developed will need to be sensitive and selective, with a wide range of detection and low limit of detection.

## Materials and methods

### Materials and reagents

Standard samples of cis-palmitoleic acid and trans-palmitoleic acid were purchased from Nu-Chek-Prep Inc. (Elysian, MN, USA), and HPLC-grade acetonitrile, n-hexane and methanol from Merck KGaA (Darmstadt, Germany). Ultrapure water was produced by a Millipore Milli-Q system (Millipore Corp., Billerica, MA, USA). The mixed crude fish oil was purchased from Xiamen Kingdomway Group Co. (Xiamen, Fujian province, China) and fresh mackerel were purchased locally in Xiamen.

### Extraction and preparation of fish oil samples

A 100-g portion of mackerel flesh was weighed and crushed then placed in a conical flask with 1 L dichloromethane. After stirring for ten h, the supernatant dichloromethane was transferred to a round flask. This procedure was repeated three times. The respective supernatants were combined, then concentrated by removing the solvent to obtain the crude fish oil.

*Using a method of saponification modified from published procedures [13–18], a 2-g sample of crude fish oil was weighed and added to 3 g potassium hydroxide, 24 mL methanol solution and 20 mL water, then stirred and heated for 2 h at 80 °C. After adjusting the pH to 3 with sulfuric acid, the mixture was centrifuged with 5000 g so that the mixed free fatty acids could be removed and concentrated by removing the solvent.

A sample of mixed free fatty acids was weighed (0.05 g), 1 mL methanol solution was added then passed through a Millex 0.2-μm nylon membrane syringe filter before analysis.

### Liquid chromatography conditions

The analytes were separated on an Agilent UPLC-1290 system (Agilent Corp., Milford, MA, USA) using a DBS C18 column (100 × 4.6 mm, 2.4 μm, Thermo Fisher Scientific, Lafayette, CO, USA). The mobile phase was composed of acetonitrile (A) and water (B), (A:B = 80:20, *v*/v) at a flow rate of 1 mL/min and the injection volume was 10 μL. The detection wavelength was set at 205 nm.

### Stock solutions and working solutions

Individual standard stock solutions of cis- and trans-POA (1.112 and 1.457 mg/mL, respectively) were prepared in acetonitrile. These stock solutions were mixed then serially diluted with acetonitrile to provide standard working solutions at six different concentrations of cis- and trans-POA from 0.05 to 500 μg/mL. All solutions were stored at − 20 °C then brought to room temperature before use.

### Method validation

The method was validated for linearity, limits of quantification and detection (LOQs and LODs), precision, repeatability, recovery and stability following the International Conference on Harmonization guidelines [19]. A series of standard solutions was prepared to determine the linearity of the analytes. Linearity was evaluated by using six calibration points in the concentration range from 0 to 500 μg/mL. The LOQ for each analyte, cis- and trans-POA, was determined at a signal-to-noise (S/N) ratio of about 3 and 10, respectively. Precision was evaluated by six injections of the standard solution within one day. Intra- and inter-day variations were chosen to determine the repeatability of the method. The mixed standard solutions were analyzed using six replicates within 24 h for intra-day variation, and for the inter-day variability test, the solutions were determined in triplicate for three consecutive days. Recovery was evaluated by spiking the samples by adding mixed standard samples at low (0.5 μg/mL), medium (5 μg/mL), and high (50 μg/mL) levels (three replicates at each concentration level) to fish oil samples, which had previously been analyzed. All variation was expressed in terms of relative standard deviation (RSD). The average recovery was estimated using the following formula:

Recovery (%) = [(detection quantity−original quantity)/quantity added] × 100.

### Determination of cis- and trans-POA by GC method

Using a modified potassium hydroxide ester exchange method [20–23], a 0.1-g sample of crude fish oil was weighed then added to 1 mL of 1 M potassium hydroxide-methanol solution (without water) and 4 mL n-hexane, heated for 30 min at 40 °C. The supernatant solution was passed through a Millex 0.2-μm nylon membrane syringe filter before injection.

The contents of cis- and trans-POA were determined using a validated GC method recently successfully established in our laboratory (Fig. 3).

### Statistical analysis

All experiments were performed at least three times. Analyses of variance (ANOVA) were performed using the Student-Newman-Keuls (S-N-K) procedure to test the significance of differences between mean values using IBM SPSS Statistics software version 23.0 (IBM Corp., Armonk, NY, USA).

## Results

### Optimization of HPLC conditions

**Fig. 2:**
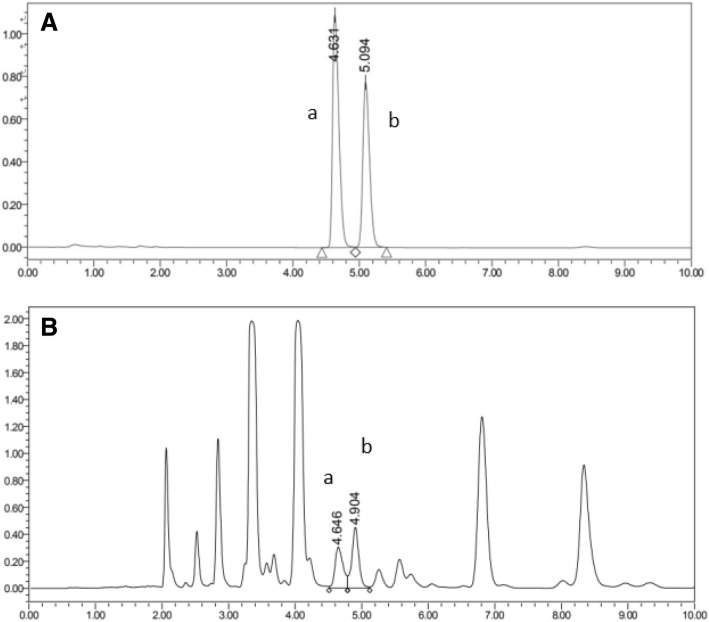
HPLC chromatogram of (A) cis- and trans-POA standard samples and (B) fish oil sample. a: cis-palmitoleic acid, b: trans-palmitoleic acid

**Fig. 3:**
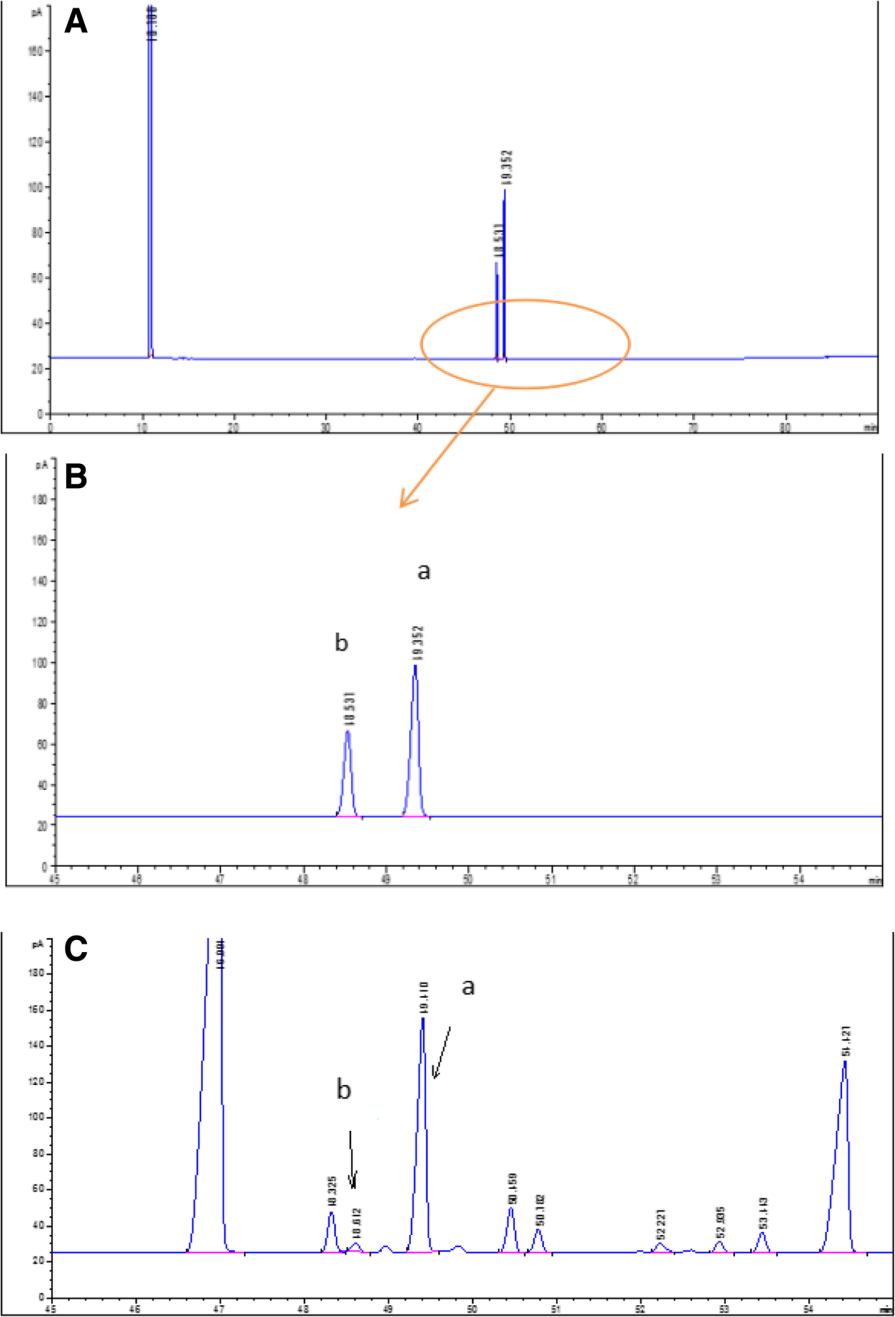
HPLC chromatogram of A, B: cis- and trans-POA standard samples and C: fish oil sample. a: cis-palmitoleic acid, b: trans-palmitoleic acid

The chromatographic conditions, in particular the analytical column, column temperature, the composition of the mobile phase, and injection volume, were optimized through several trials to obtain chromatograms with a satisfactory resolution, appropriate retention times, and high sensitivity. The BDS C18 column was selected as it produced satisfactory separations and peak shapes with good resolution. Various mobile phases, such as methanol or acetonitrile and water, at different solvent ratios (60:40, 70:30, 80:20, 90:10), were tested for their sensitivity and good chromatographic behavior. The results showed that using a mobile phase of acetonitrile and water (80:20) was most efficient and produced symmetrical peaks for both analytes. The peak times and run times for determining cis- and trans-POA using the HPLC method of 4.6–5.1 min, and 10 min, respectively, (Fig. 2) were much shorter than those using the GC method of 48.3–49.1 min, and 90 min, respectively (Fig. 3).

### Validation of the analysis method

Standard curves were established by plotting the ratios of the chromatogram peak areas for cis- and trans-POA. These curves exhibited correlation coefficients greater than 0.999 and good linearity over concentration ranges from 0.5 to 500 μg/mL for cis- and trans-POA. Typical calibration equations were:

y = 3012x - 831.27 (r = 0.9991) for cis-POA, and.

y = 3949.6x + 58.628 (r = 0.9999) for trans-POA,

where y represents the peak area ratio of an analyte, and x represents an analyte concentration.

The lower limits of quantification (LLOQ) for cis- and trans-POA were defined as 0.5 and 0.2 μg/mL, respectively, based on a signal-to-noise ratio (S/N) of 10. The limit of detection (LOD) was estimated to be 0.2 and 0.05 μg/mL, respectively, based on an S/N of 3.

The results for precision, repeatability, stability and recovery of cis and trans-POA are summarized in Tables 1 and 2. The mean relative standard deviation (RSD) values for intra-day and inter-day variation were 1.26 and 0.96% for cis-POA, and 1.10 and 0.63% for trans-POA, respectively. The HPLC method also had an acceptable accuracy with an average recovery of 94.12 and 103.61% for cis- and trans-POA, respectively. This demonstrated that the HPLC method developed was sufficiently reliable and accurate to be suitable for quantifying cis- and trans-POA in fish oil.

**Table 1:** Precision, repeatability and stability for determination of cis and trans-POA contents of fish oil using HPLC

Analyte	Precision (RSD, %, *n* = 6)	Repeatability (RSD, %, *n* = 6)				Stability (RSD, %, *n* = 6)
		Day 1	Day 2	Day 3	Inter-day	
Cis-POA	1.48	1.26	0.89	1.25	0.96	1.05
Trans-POA	0.98	0.57	0.63	1.10	0. 63	0.79

**Table 2:** Recovery of cis and trans-POA for determination of cis and trans-POA contents of fish oil using HPLC

Analyte	Low	Medium	High	Average
	Recovery±RSD (%)	Recovery±RSD (%)	Recovery±RSD (%)	Recovery±RSD (%)
Cis-POA	93.37 ± 3.12	94.77 ± 3.31	94.62 ± 1.43	94.12 ± 3.26
Trans-POA	98.72 ± 2.92	105.91 ± 2.63	106.96 ± 1.61	103.61 ± 2.76

### Sample analysis

The HPLC and GC methods were successfully applied to the quantification of cis- and trans-POA in fish oil, and the results are summarized in Table 3. The results reveal significant variation in the contents of the cis- and trans-POA quantified in fish oil by both the HPLC and GC methods. In particular, the contents of trans-POA in the mackerel and mixed fish oils were 6.49, and 6.73%, respectively, using HPLC and 0.41, and 0.21%, respectively, using GC. Such variation may have been caused by incomplete methyl esterification. After repeated verification and validation using standard samples, it was found that triglyceride-POA, resulting from the potassium hydroxide ester exchange method, allowed easy methylation but when cis- and trans-POA were in the free acid state they could hardly be methylated.

**Table 3:** The content of cis- and trans-POA in crude fish oil

	Content (%) in crude fish oil			
	Extracted from Mackerel fish		Purchased (Mixed fish oil)	
	Cis-POA	Trans-POA	Cis-POA	Trans-POA
HPLC method	7.93	6.49	5.26	6.73
GC method	7.54	0.41	4.26	0.21

## Discussion

HPLC and GC methods have been used extensively for half a century for lipid analysis [20–22]. Although GC has been used most widely for analyzing and determining fatty acids, the application of HPLC has increased recently with both methods having characteristic strengths and weaknesses. Usually fatty acid samples have been detected and quantified by the GC method. To reduce the residue of fatty acids in the chromatographic column caused by burning at high temperature, the fatty acids have been almost completely methyl esterified, a crucial process for achieving high levels of accuracy and precision of determination.

Current methods for methyl esterification include potassium hydroxide ester exchange [20, 21], the use of hydrochloric acid [22, 24], or boron trifluoride [25–27] and the methyl sulfate method [23]. Free fatty acids in the glyceride state are hardly methyl esterified using the potassium hydroxide ester exchange method but fatty acids in the glyceride state are easily methyl esterified. However, the boron trifluoride methyl esterification method has been reported as insufficient for derivatizing both free fatty acids and fatty acids in the glyceride state [23]. In contrast, methanolic hydrochloric acid (HCl) as well as a combination of BF_3_ with methanolic sodium hydroxide (NaOH+BF_3_) has been reported as suitable for the derivatization of free fatty acids, polar lipids, and triglycerides (derivatization rate > 80% for all tested lipids) [23].

In the present study, the potassium hydroxide ester exchange method was chosen to derivatize the cis- and trans-POA. It was found that the results from the HPLC and GC methods were different, because the free fatty acids were not derivatized in the presence of potassium hydroxide, a result agreeing with Ostermann et al. [23]. It was suggested that cis-POA may be in the glyceride state, while the trans-POA was almost completely present as a free fatty acid in natural fish oil, so that the derivatization rate using any method of methyl esterification with cis- and trans-POA in fish oil would be different. Therefore, the HPLC method was considered to be more suitable for analyzing cis- and trans-POA in the free fatty acids state simultaneously by saponification. Thus, the HPLC method would be simpler and more accurate than GC, because it does not require the derivatization step.

The presence of cis- and trans-POA in natural fish oil in different states or forms is related to the differences in their biological activity. This study provides technical support for the study of these pharmacological differences and the relationship between the structure and activity of cis- and trans-POA. With its lower limits of quantification and wider range of quantification, the HPLC method has been shown to be suitable for detecting many types of compound, such as palmitoleic acid-methyl ester, palmitoleic acid-ethyl ester, and palmitoleic acid-glyceride, simultaneously. This method could also be used to analyze cis- and trans-POA in plasma or other biological tissue, to help physicians analyze patients’ samples quickly, in 10 min, and therefore provide a more rapid diagnosis of problems relating to the risk of type II diabetes and coronary heart disease.

## Conclusions

A highly sensitive, reproducible and accurate HPLC method has been developed for comprehensively determining cis- and trans-POA contents in fish oil. The contents of the quantified cis-POA were similar using the HPLC and GC methods, but those of trans-POA were significantly different. A comprehensive consideration of the characteristics of saponification and methyl esterification suggested that the HPLC method was more suitable for determining cis- and trans-POA contents in fish oil, and that in natural fish oil, cis-POA may be in the glyceride state while trans-POA was a free fatty acid.

## Abbreviations

Cis-POA: Cis-palmitoleic acid
GC: Gas chromatography
HPLC: High-pressure liquid chromatography
POA: Palmitoleic acid
Trans-POA: Trans-palmitoleic acid
